# *H19* overexpression promotes leukemogenesis and predicts unfavorable prognosis in acute myeloid leukemia

**DOI:** 10.1186/s13148-018-0486-z

**Published:** 2018-04-10

**Authors:** Ting-juan Zhang, Jing-dong Zhou, Wei Zhang, Jiang Lin, Ji-chun Ma, Xiang-mei Wen, Qian Yuan, Xi-xi Li, Zi-jun Xu, Jun Qian

**Affiliations:** 1 Department of Hematology, Affiliated People’s Hospital of Jiangsu University, 8 Dianli Rd., Zhenjiang, 212002 Jiangsu People’s Republic of China; 2 School of Medicine, Jiangsu University, Zhenjiang, Jiangsu People’s Republic of China; 3 The Key Lab of Precision Diagnosis and Treatment of Zhenjiang City, Zhenjiang, Jiangsu People’s Republic of China; 4 Laboratory Center, Affiliated People’s Hospital of Jiangsu University, Zhenjiang, Jiangsu People’s Republic of China

**Keywords:** *H19*, Prognosis, Surveillance, *ID2*, AML

## Abstract

**Background:**

The long non-coding RNA *H19* plays a crucial role in solid tumor initiation and progression. However, the potential role of *H19* and its clinical significance in acute myeloid leukemia (AML) remain largely elusive.

**Methods:**

*H19* expression was detected by qPCR, and clinical significance in AML patients was further analyzed. The Cancer Genome Atlas (TCGA) and Gene Expression Omnibus (GEO) data for AML were used as validation cohorts. The roles of *H19* in cell proliferation and apoptosis were determined by cell proliferation assay and flow cytometry analysis.

**Results:**

*H19* expression was significantly increased in AML patients but not associated with embedded *miR-675* expression. Moreover, *H19* overexpression was not dependent on the methylation pattern in *H19* differentially methylated region/imprinting control region. Strong association was observed between *H19* overexpression and patients’ characteristics including sex, higher white blood cells, older age, and intermediate karyotype, *FLT3*-ITD, and *DNMT3A* mutations. In addition, *H19* overexpression correlated with lower complete remission (CR) rate and shorter overall survival, and further confirmed by multivariate analyses. Importantly, the prognostic effect of *H19* expression was validated by TCGA and GEO data. In the follow-up of patients, *H19* expression in CR phase was lower than diagnosis time and returned at relapse time. Loss-of-function experiments showed that *H19* exhibited anti-proliferative and pro-apoptotic effects in leukemic cell HL60. Furthermore, *H19* expression was positively correlated with potential downstream gene *ID2* in AML.

**Conclusions:**

Our findings revealed that methylation-independent *H19* was a prognostic and predictive biomarker in AML, and *H19*/*ID2* played crucial roles in leukemogenesis with potential therapeutic target value.

**Electronic supplementary material:**

The online version of this article (10.1186/s13148-018-0486-z) contains supplementary material, which is available to authorized users.

## Background

Acute myeloid leukemia (AML), the most common adult leukemia, is an etiologically, clinically, cytogenetically, and molecularly heterogeneous disease characterized by uncontrolled proliferation and blocked apoptosis of immature myeloid progenitors [1]. Genetic abnormalities and epigenetic alterations played crucial roles in the pathogenesis of AML [2]. Moreover, genetic abnormalities such as chromosome aberrations and gene mutations were also seen as the most powerful prognostic information [3]. Despite recent advances in the anti-cancer or targeted drugs, clinical outcome of AML remains unsatisfactory [1]. Accordingly, progresses should be made in the mechanisms of leukemogenesis and the identification of markers that allow molecular-based stratification to risk-adapted therapies to improve the clinical outcome of AML.

Recently, long non-coding RNAs (lncRNAs) have been implicated in many human diseases especially in human cancers, and increasing studies begin to unravel the molecular mechanisms underlying lncRNA function in these pathological processes and/or carcinogenesis [4]. The human *H19* gene encodes a 2.3-kb lncRNA with a crucial role in embryonal development and growth control [5]. *H19* and neighboring gene *IGF2* (known as *IGF2*/*H19* locus) are reciprocally imprinted, leading to differential allelic expression of *H19* from the maternal allele and *IGF2* from the paternal allele [6]. Abnormal expression or loss of imprinting of *H19* has also been linked to diverse human cancers including hematological malignancies [5]. Although *H19* was originally seen as a tumor suppressor in Wilms’ tumors, embryonic rhabdomyosarcoma, and Beckwith-Wiedmann cancer predisposing syndrome, recent studies displayed the evidences of the oncogenic role of *H19* in several human cancers, such as breast cancer, endometrial cancer, gastric cancer, and so on [5, 7]. Notably, Guo et al. reported that high expression of *H19* was required for efficient tumorigenesis induced by *BCR-ABL* oncogene [7]. In addition, loss of imprinting (LOI) of *IGF2/H19* mainly caused by “differentially methylated region” or “imprinting control region” (DMR/ICR) demethylation was shown as a frequent event in AML, adult T cell leukemia/lymphoma, and chronic myeloid leukemia (CML) [8–10]. However, the direct role and its clinical significance in AML remain poorly determined. Herein, we reported *H19* as a prognostic and predictive biomarker in AML, and *H19* played a crucial role in leukemogenesis with potential therapeutic target value.

## Methods

### Patients and treatment

A total of 161 AML patients [including 161 newly diagnosed patients, 54 patients who achieved complete remission (CR) after induction therapy and 26 relapsed patients] and 36 healthy donors were included in this study approved by the Institutional Ethics Committee of the Affiliated People’s Hospital of Jiangsu University. After written informed consents were obtained, bone marrow (BM) was collected from all participants and was extracted for the BM mononuclear cells (BMMNCs). All the patients received induction and consolidation chemotherapy as reported in our previous literature [11].

### Cytogenetic analysis and gene mutation detection

Karyotypes were analyzed at the newly diagnosis time by conventional R-banding method according to the previous literature [12]. Gene mutations (such as *NPM1* and *DNMT3A* mutations) were detected by high-resolution melting analysis (HRMA) and direct DNA sequencing (such as *CEBPA* and *FLT3*-ITD mutations) as reported [13–21]*.*

### RNA isolation, reverse transcription, and RT-qPCR

Total RNA was isolated using Trizol reagent (Invitrogen, Carlsbad, CA, USA) and was transcriptionally reversed into cDNA as reported previously [22]. *H19* expression was detected by real-time quantitative PCR (RT-qPCR) using the SYBR Premix Ex Taq II (TaKaRa, Tokyo, Japan) with the primers shown in Additional file 1: Table S1. The RT-qPCR reaction was carried out at 95 °C for 30 s, followed by 40 cycles at 95 °C for 5 s, 67 °C for 30 s, 72 °C for 30 s, and 87 °C for 30 s to collect fluorescence. *ABL1* expression was detected by RT-qPCR using AceQ qPCR SYBR Green Master Mix (Vazyme Biotech Co., Piscataway, NJ, USA) as reported [22]. Relative *H19* level was calculated using the following equation: N_*H19*_ = (E_*H19*_) ^ΔCT *H19* (control − sample)^ ÷ (E_*ABL*_) ^ΔCT *ABL* (control − sample)^. The parameter efficiency (*E*) was derived from the formula *E* = 10^(−1/slope)^ (the slope referred to CT versus cDNA concentration plot).

### DNA isolation, chemical modification, and RT-qMSP

Genomic DNA was isolated and modified using genomic DNA purification kit (Gentra, Minneapolis, MN, USA) and CpGenome™ DNA Modification Kit (Chemicon, Ternecula, Canada), respectively. The level of *H19* DMR/ICR methylation was detected by the unmethylation primers (Additional file 1: Table S1) of real-time quantitative methylation-specific PCR (U-RT-qMSP) with SYBR Premix Ex Taq II (TaKaRa, Tokyo, Japan). U-RT-qMSP conditions were 95 °C for 30 s and 40 cycles for 5 s at 95 °C, 30 s at 57 °C, 30 s at 72 °C, and 75 °C for 30 s. The normalized ratio (N_*U-H19*_) was applied to assess the level of *H19* unmethylation in samples. N_*U-H19*_ was calculated using the following formula: N_*U-H19*_ = (E_*U-H19*_) ^ΔCT *U-H19* (control − sample)^ ÷ (E_*ALU*_) ^ΔCT *ALU* (control − sample)^.

### Bisulfite sequencing PCR

Bisulfite sequencing PCR (BSP) reaction was carried out using TaKaRa Taq™ Hot Start Version kit (Tokyo, Japan) as reported [11]. The main conditions were 98 °C for 10 s, 55 °C for 30 s, and 72 °C for 30 s. Nine independent clones per specimen were picked out and sequenced.

### Cell line and cell culture

Human leukemic cell line HL60 (American Type Culture Collection, Manassas, VA, USA) was cultured in RPMI 1640 medium (BOSTER, Wuhan, China) containing 10% fetal calf serum (ExCell Bio, Shanghai, China) and grown at 37 °C in 5% CO_2_ humidified atmosphere.

### SiRNA transfection

SiRNA-mediated knockdown of *H19* was used for loss-of-function experiments. The si*H19*-1 (sense strand: 5′-CCCGUCCCUUCUGAAUUUATT-3′; antisense strand: 5′-UAAAUUCAGAAGGGACGGGTT-3′) and si*H19*-2 (sense strand: 5′-UAAGUCAUUUGCACUGGUUTT-3′; antisense strand: 5′-AACCAGUGCAAAUGACUUATT-3′) [23] were purchased from GenePharma (Shanghai, China). SiRNA transfection was performed using the X-tremeGENE siRNA Transfection Reagent (Roche, Basel, Switzerland) according to the manufacturer’s instructions. Cells were used for experiments in 3 days after siRNA transfection.

### Cell proliferation assays

Cells (1 × 10^5^ cells/mL) for 2 mL per well were seeded in a 6-well plate in RPMI 1640 medium containing 10% fetal calf serum. After culturing for 0, 1, 2, and 3 days, cells were counted in a counting board for three times.

### Cell apoptosis assays

Cells (2 × 10^5^ cells/mL) for 2 mL per well were seeded in a 6-well plate in RPMI 1640 medium containing 0% fetal calf serum. Annexin V-PE/7-AAD apoptosis detection (BD Pharmingen, San Diego, CA, USA) was used and then analyzed via flow cytometry (BD FACSCalibur, San Jose, CA, USA). Each experiment was repeated three times.

### TCGA and GEO datasets

*H19* expression (RNA Seq V2 RSEM) and *H19* methylation (HM27 and HM450) data from a cohort of 200 AML patients from The Cancer Genome Atlas (TCGA) [24] were downloaded via cBioPortal (http://www.cbioportal.org) [25, 26].

Two independent cohorts of 78 and 162 cytogenetically normal AML (CN-AML) patients from Gene Expression Omnibus (GEO) data (http://www.ncbi.nlm.nih.gov/geo/; accession number GSE12417) were applied to analyze the prognostic impact of *H19* expression using the online web tool Genomicscape (http://genomicscape.com/microarray/survival.php) [27, 28].

### Bioinformatics analyses

*H19* function prediction based on text mining was performed using the Coremine Medical online database (http://www.coremine.com/medical/).

### Statistical analyses

SPSS 20.0 software package and GraphPad Prism 5 were applied to statistical analyses. Mann-Whitney’s *U* test was performed to compare the differences of continuous variables, whereas the differences of categorical variables were analyzed using the Pearson chi-square analysis/Fisher exact test. Spearman correlation test was conducted to evaluate the correlation between continuous variables. The ROC curve and area under the ROC curve (AUC) were carried out to assess the discriminative capacity of *H19* expression between patients and controls. *H19* expression for achievement of CR was evaluated via logistic regression models (univariate and multivariate). Kaplan-Meier and Cox regression (univariate and multivariate) analyses were used to analyze the impact of *H19* expression on overall survival (OS) and leukemia-free survival (LFS). Statistical significance was set at *P* < 0.05 and all tests were two sided.

## Results

### *H19* expression was upregulated in AML

**Fig. 1:**
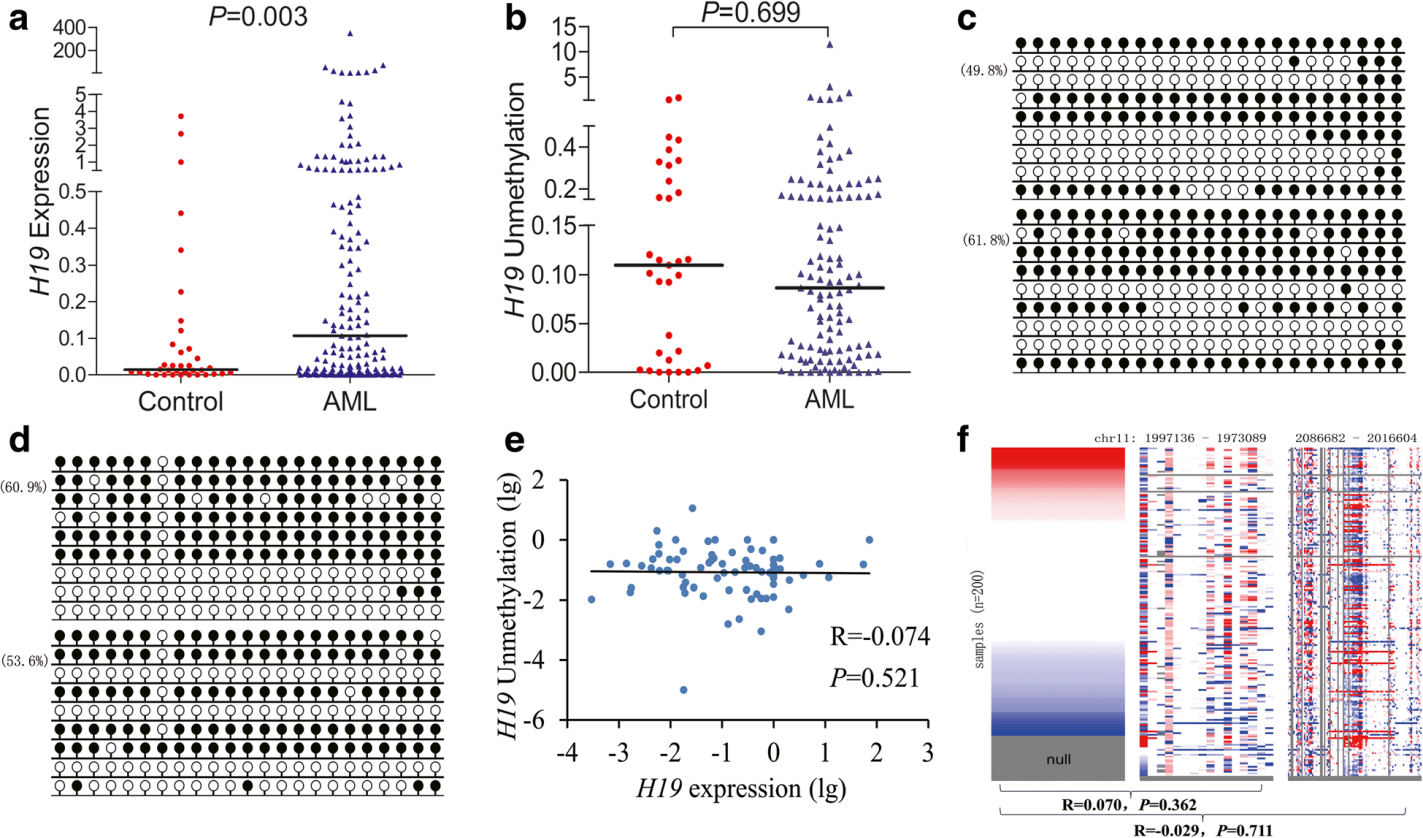
*H19* expression and methylation in AML. **a** *H19* expression level detected by real-time quantitative PCR in controls and AML patients. **b** *H19* unmethylation level detected by real-time quantitative unmethylation-specific PCR in controls and AML patients. **c**, **d** *H19* methylation density detected by bisulfite sequencing in controls and AML patients, respectively. White cycle, unmethylated CpG dinucleotide; black cycle, methylated CpG dinucleotide. **e** The correlations between *H19* expression and unmethylation. **f** Relationship between *H19* expression and methylation of two different regions using The Cancer Genome Atlas (TCGA) data. Left: *H19* expression, value in log2(*x* + 1) transformation, *x* is the RSEM value. Middle: *H19* methylation in HM27K. Right: *H19* methylation in HM450K. Red color: higher expression/methylation. Blue color: lower expression/methylation. White color: intermediate level of *H19* expression/methylation. Gray color: no data. Each row represents *H19* expression and *H19* methylation level of the same patient; each line from right to left in middle and right figures represents the methylation level at different sites

In order to determine the role of *H19* in AML pathogenesis, we first evaluated *H19* expression in AML patients and controls by RT-qPCR. As presented in Fig. 1a, *H19* expression was significantly upregulated in AML patients (median 0.107) than controls (median 0.014) (*P* = 0.003). Since *microRNA-675* (*miR-675*) is embedded within the first exon of *H19*, we further assess the association of *H19* with *miR-675* in AML. Previously, our study reported *miR-675* expression was significantly downregulated in AML patients [29]. Herein, we further found that there was no significant correlation between *H19* and *miR-675* expression in AML (*R* = 0.032, *P* = 0.750, *n* = 101).

### *H19* overexpression was not dependent on H19 methylation in AML

Since *H19* is an imprinted gene and controlled by the methylation pattern in DMR/ICR, we hypothesized that *H19* overexpression was mediated by *H19* DMR/ICR hypomethylation in AML. However, RT-qMSP showed that its DMR/ICR methylation level in AML patients (median 0.086) was similar to controls (median 0.109) (*P* = 0.699, Fig. 1b). The same result was also confirmed by BSP analysis (Fig. 1c, d). Moreover, no significant association was observed between *H19* DMR/ICR methylation and expression in AML (*R* = − 0.074, *P* = 0.521, *n* = 77, Fig. 1e).

In order to verify our results, we further implemented an independent assessment of *H19* methylation and expression in AML from TCGA database. As expected, no significant negative correlation was observed between *H19* methylation and expression in AML (*R* = 0.070, *P* = 0.362, *n* = 170 and *R* = − 0.029, *P* = 0.711, *n* = 170, respectively, Fig. 1f).

### *H19* overexpression correlated with clinical characteristics and genetic events in AML

ROC curve analysis revealed that the sensitivity and the specificity were 49.1 and 80.6% (sensitivity + specificity − 1 was the highest value) when *H19* expression was at the value of 0.121 (Additional file 2: Figure S1). By the cutoff value, we classified the whole-cohort AML patients into two groups (high and low) in order to further analyze the clinical significance of *H19* expression in AML. High *H19* expression was found to be associated with sex (*P* = 0.075), higher white blood cells (*P* = 0.009), and older age (*P* = 0.004, Table 1). Moreover, significant differences were observed among both karyotype and karyotypic classifications (*P* = 0.048 and 0.010, respectively). *H19* overexpression had the highest frequency in intermediate karyotype [70% (55/79), *P* = 0.002] and much lower in favorable karyotype [18% (14/79), *P* = 0.013] especially in t(15;17) [6% (5/79), *P* = 0.008].

**Table 1:** Comparison of clinical manifestations and laboratory features between *H19*^low^ and *H19*^high^ AML patients

Patient’s parameters	Low (*n* = 82)	High (*n* = 79)	*P* value
Sex, male/female	44/38	54/25	0.075
Median age, years (range)	51.5 (10–87)	61 (17–93)	0.009
Median WBC, × 10^9^/L (range)	7.7 (1.0–185.4)	31.1 (0.3–528.0)	0.004
Median hemoglobin, g/L (range)	75 (40–133)	78.5 (32–144)	0.144
Median platelets, × 10^9^/L (range)	42 (5–447)	33 (3–399)	0.262
Median BM blasts, % (range)	44 (3.0–94.5)	43 (1–99)	0.339
Karyotype classification			0.010
Favorable	29 (35%)	14 (18%)	
Intermediate	37 (45%)	55 (70%)	
Poor	12 (15%)	9 (11%)	
No data	4 (5%)	1 (1%)	
Karyotype			0.048
Normal	28 (34%)	42 (54%)	
t(8;21)	7 (9%)	5 (6%)	
t(16;16)	0 (0%)	1 (1%)	
t(15;17)	22 (27%)	8 (10%)	
t(9;22)	0 (0%)	1 (1%)	
+ 8	3 (4%)	4 (5%)	
− 5/5q−	1 (1%)	2 (3%)	
− 7/7q−	1 (1%)	0 (0%)	
Complex	10 (12%)	6 (8%)	
Others	6 (7%)	9 (11%)	
No data	4 (5%)	1 (1%)	
Gene mutation			
*CEBPA* (+/−)	10/66	7/61	0.617
*NPM1* (+/−)	6/70	11/57	0.195
*FLT3*-ITD (+/−)	6/70	13/55	0.053
*c-KIT* (+/−)	4/72	1/67	0.370
*N/K-RAS* (+/−)	4/72	8/60	0.228
*IDH1/2* (+/−)	2/74	6/62	0.149
*DNMT3A* (+/−)	2/74	9/59	0.025
*U2AF1* (+/−)	3/73	3/65	1.000
*SRSF2* (+/−)	3/75	4/66	0.708
*SETBP1* (+/−)	1/77	1/69	1.000
CR (+/−)	40/35	22/52	0.005

*AML* acute myeloid leukemia, *WBC* white blood cells, *CR* complete remission

We further assessed the association of *H19* expression with gene mutations in AML. A total of 12 common gene mutations were screened in 144 AML patients. Patients with high *H19* expression harbored higher incidence of *FLT3*-ITD and *DNMT3A* mutations than those with low *H19* expression (*P* = 0.053 and 0.025, respectively, Table 1). No significant differences were observed in other gene mutations among the two groups (*P* > 0.05, Table 1).

### *H19* overexpression correlated with poor chemotherapy response and OS in AML

Follow-up data was available for 149 AML patients including 121 non-APL-AML patients and 64 CN-AML patients. As shown in Table 1, whole-cohort AML patients with high *H19* expression had a significantly lower CR rate than those with low *H19* expression (*P* = 0.005). The similar results also existed among non-APL-AML and CN-AML patients (*P* = 0.012 and 0.036, respectively). Moreover, multivariate analysis revealed that high *H19* expression taken as a dichotomous variable was an independent prognostic predictor for poor CR rate among both whole-cohort and non-APL-AML patients (*P* = 0.034 and 0.011, respectively, Table 2) but not CN-AML patients (data not shown).

**Table 2:** Univariate and multivariate analyses of prognostic factors for complete remission in whole-cohort and non-APL-AML patients

	Univariate analysis		Multivariate analysis	
	Odds ratio (95% CI)	*P* value	Odds ratio (95% CI)	*P* value
Whole-cohort AML				
Age	0.119 (0.054–0.262)	< 0.001	0.162 (0.069–0.379)	< 0.001
WBC	0.269 (0.126–0.575)	0.001	0.505 (0.204–1.253)	0.140
Karyotype classifications	0.214 (0.112–0.408)	< 0.001	0.269 (0.139–0.519)	< 0.001
*H19* expression	0.370 (0.189–0.726)	0.004	0.416 (0.185–0.935)	0.034
Non-APL AML				
Age	0.164 (0.069–0.392)	< 0.001	0.199 (0.079–0.502)	0.001
WBC	0.353 (0.153–0.819)	0.015	0.522 (0.198–1.378)	0.189
Karyotype classifications	0.316 (0.145–0.691)	0.004	0.297 (0.129–0.687)	0.005
*H19* expression	0.361 (0.164–0.791)	0.011	0.306 (0.123–0.762)	0.011

Variables were composed of age (≤ 60 vs. > 60 years), WBC (≥ 30 × 10^9^ vs. < 30 × 10^9^/L), karyotype classifications (favorable vs. intermediate vs. poor), and *H19* expression (low vs. high). The multivariate analysis included variables with *P* < 0.100 in univariate analysis for complete remission

*AML* acute myeloid leukemia, *APL* acute promyelocytic leukemia, *WBC* white blood cells, *CI* confidence interval

**Fig. 2:**
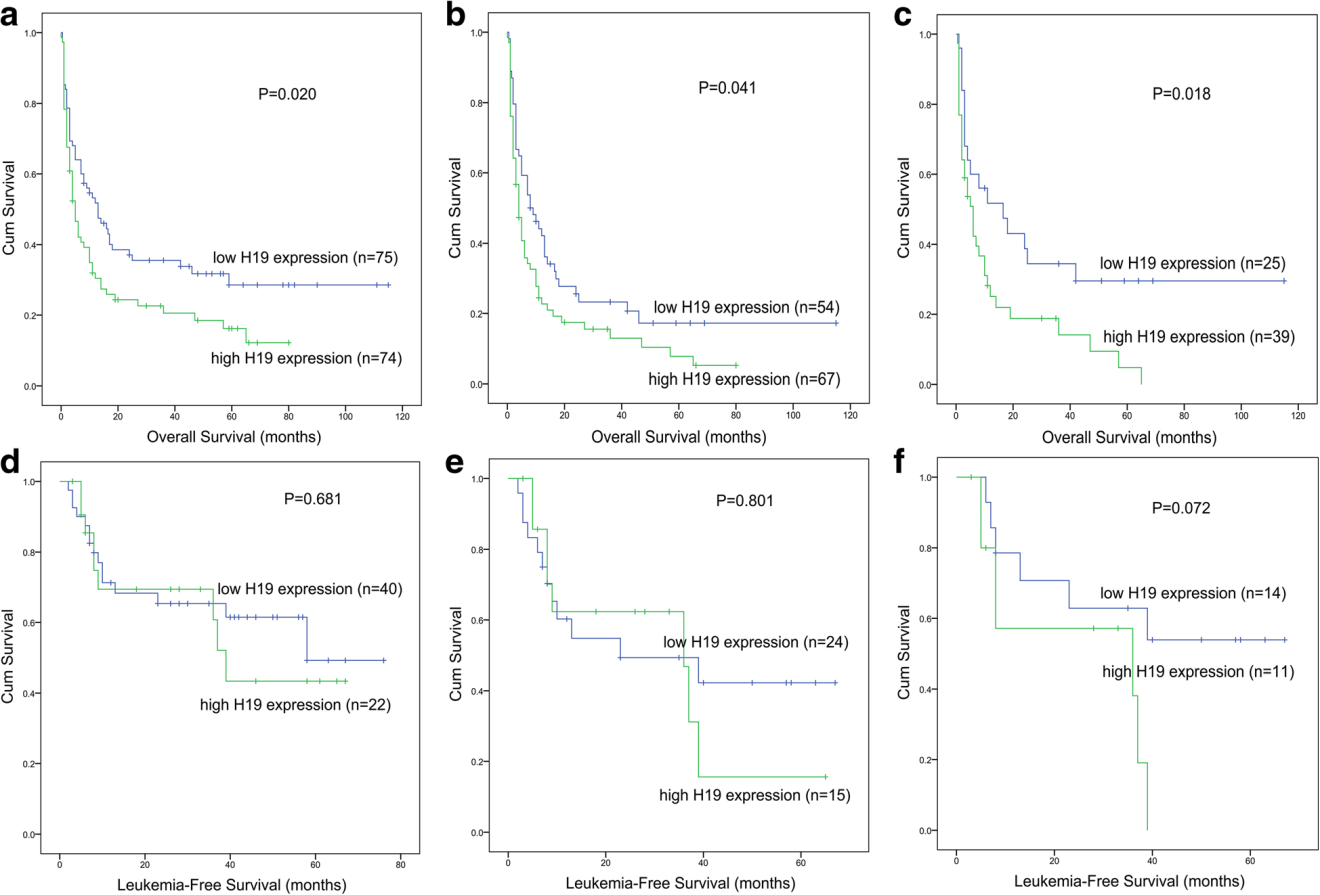
The impact of *H19* expression on survival in AML. **a** Overall survival (OS) among whole-cohort AML patients. **b** OS among non-APL-AML patients. **c** OS among cytogenetically normal AML (CN-AML) patients. **d** Leukemia-free survival (LFS) among whole-cohort AML patients. **e** LFS among non-APL-AML patients. **f** LFS among CN-AML patients

Kaplan-Meier analysis revealed that whole-cohort AML patients with *H19* overexpression had a significantly shorter OS than those without *H19* overexpression (*P* = 0.020, Fig. 2a). Among non-APL-AML and CN-AML, patients with high *H19* expression were also associated with shorter OS (*P* = 0.041 and 0.018, Fig. 2b, c, respectively). However, there was no significant association between *H19* expression and LFS among either AML sub-groups (all *P* > 0.05, Fig. 2d–f, respectively). Since *H19* expression closely correlated with several well-established prognostic factors such as age, WBC, karyotypic classifications, and gene mutations, we further conducted a Cox regression model adjusting for prognosis-related factors. Multivariate analysis revealed that high *H19* expression might act as an independent prognostic biomarker for poor OS in non-APL-AML patients (HR = 1.554, *P* = 0.063, Table 3) but not whole-cohort AML (HR = 1.355, *P* = 0.169) or CN-AML patients (HR = 1.393, *P* = 0.313).

**Table 3:** Univariate and multivariate analyses of prognostic factors for overall survival in non-APL-AML patients

	Univariate analysis		Multivariate analysis	
	Hazard ratio (95% CI)	*P* value	Hazard ratio (95% CI)	*P* value
Age	2.294 (1.528–3.446)	< 0.001	1.651 (1.067–2.555)	0.024
WBC	1.856 (1.247–2.764)	0.002	1.447 (0.943–2.220)	0.091
Karyotype classifications	1.756 (1.314–2.346)	< 0.001	1.858 (1.320–2.616)	< 0.001
*H19* expression	1.486 (0.997–2.216)	0.052	1.554 (0.977–2.472)	0.063
*CEBPA* mutation	0.793 (0.410–1.533)	0.491		
*NPM1* mutation	1.142 (0.606–2.152)	0.681		
*FLT3*-ITD mutation	1.005 (0.532–1.898)	0.987		
*c-KIT* mutation	1.043 (0.255–4.263)	0.953		
*N/K-RAS* mutation	1.070 (0.533–2.149)	0.849		
*IDH1/2* mutation	4.246 (1.964–9.179)	< 0.001	3.781 (1.593–8.978)	0.003
*DNMT3A* mutation	1.256 (0.630–2.506)	0.518		
*U2AF1* mutation	2.756 (1.177–6.455)	0.020	2.499 (1.050–5.950)	0.038
*SRSF2* mutation	2.005 (0.914–4.399)	0.083	1.590 (0.673–3.758)	0.291
*SETBP1* mutation	0.497 (0.069–3.583)	0.488		

Variables were composed of age (≤ 60 vs. > 60 years), WBC (≥ 30 × 10^9^ vs. < 30 × 10^9^/L), karyotype classifications (favorable vs. intermediate vs. poor), *H19* expression (low vs. high), and gene mutations (mutant vs. wild-type). The multivariate analysis included variables with *P* < 0.100 in univariate analysis for overall survival

*AML* acute myeloid leukemia, *APL* acute promyelocytic leukemia, *WBC* white blood cells, *CI* confidence interval

### The prognostic value of *H19* expression validated by TCGA and GEO data

**Fig. 3:**
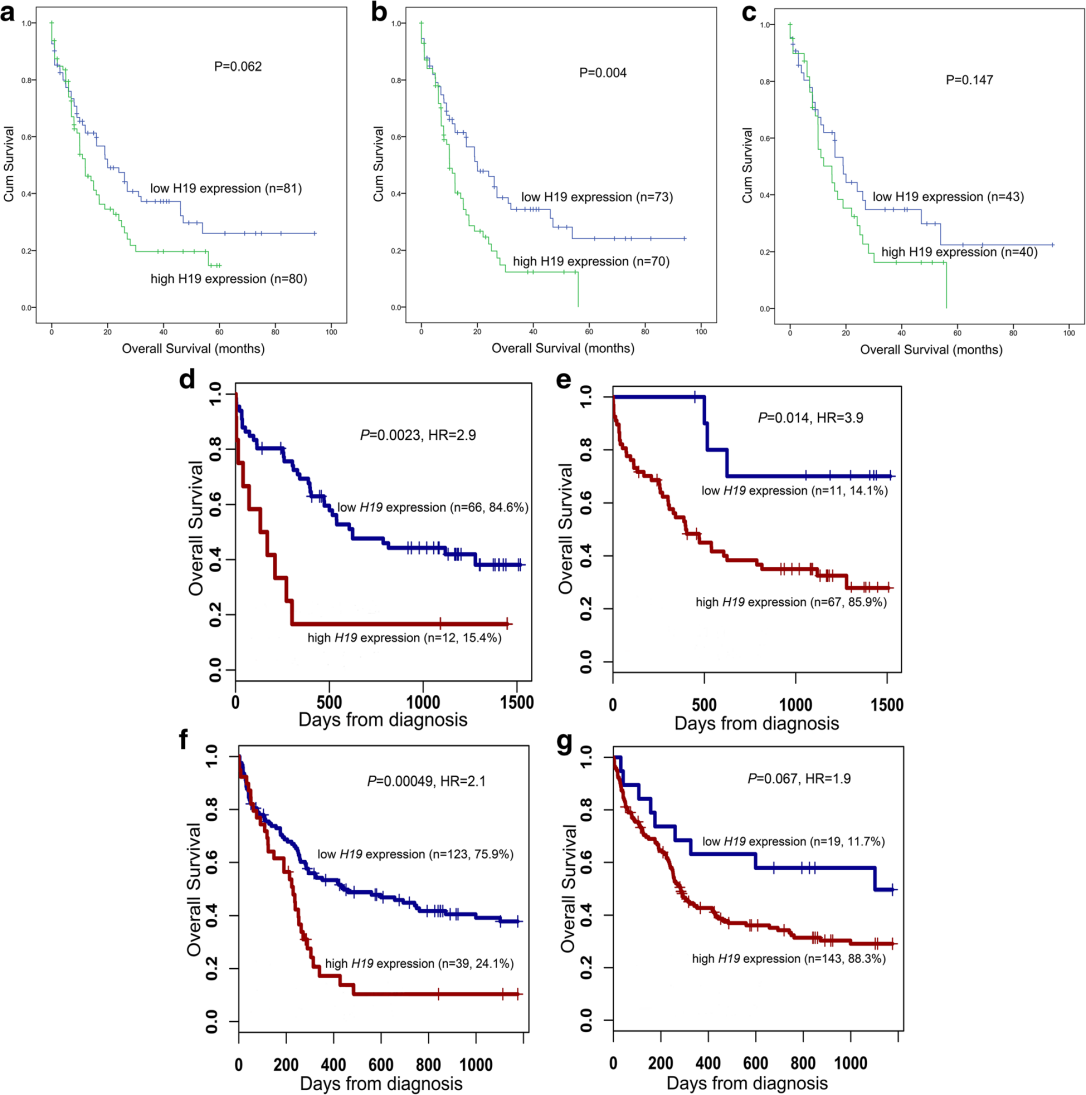
Prognostic value of *H19* expression on overall survival in AML using TCGA and GEO data. **a**–**c** The impact of *H19* expression on overall survival (OS) in a cohort of 200 AML patients from The Cancer Genome Atlas (TCGA) databases. The patients were classified into *H19* low-expressed and high-expressed groups by the median level of *ID4* expression. **a** OS among whole-cohort AML. **b** OS among non-APL-AML. **c** OS among cytogenetically normal AML (CN-AML). **d**–**g** The impact of *H19* expression on OS in two independent cohorts of 78 and 162 CN-AML patients were obtained from Gene Expression Omnibus (GEO) data. Survival analysis was performed through the online web tool Genomicscape. **d** Probe 224646_at among a cohort of 78 CN-AML patients. **e** Probe 224997_at among a cohort of 78 CN-AML patients. **f** Probe 224646_at a cohort of 162 CN-AML patients. **g** Probe 224997_at among a cohort of 162 CN-AML patients

In order to validate the prognostic value of *H19* expression in AML, we searched and analyzed an independent assessment in AML patients from TCGA databases. By the median level of *H19* expression set as the cut-off value, patients with higher *H19* expression showed a significantly shorter OS among both whole-cohort AML (*P* = 0.062, Fig. 3a) and non-APL-AML (*P* = 0.004, Fig. 3b). Nevertheless, no significant difference was observed between the two groups for OS among CN-AML (*P* = 0.147, Fig. 3c).

Moreover, the published data from two cohorts of CN-AML patients available in GEO databases were set as the independent validation cohort. Through the online tool GenomicScape, high *H19* expression was significantly correlated with shorter OS among both two cohorts (*P* = 0.002, 0.014, < 0.001, and 0.067, respectively, Fig. 3d–g).

### *H19* expression was a predictive biomarker in the surveillance of AML

**Fig. 4:**
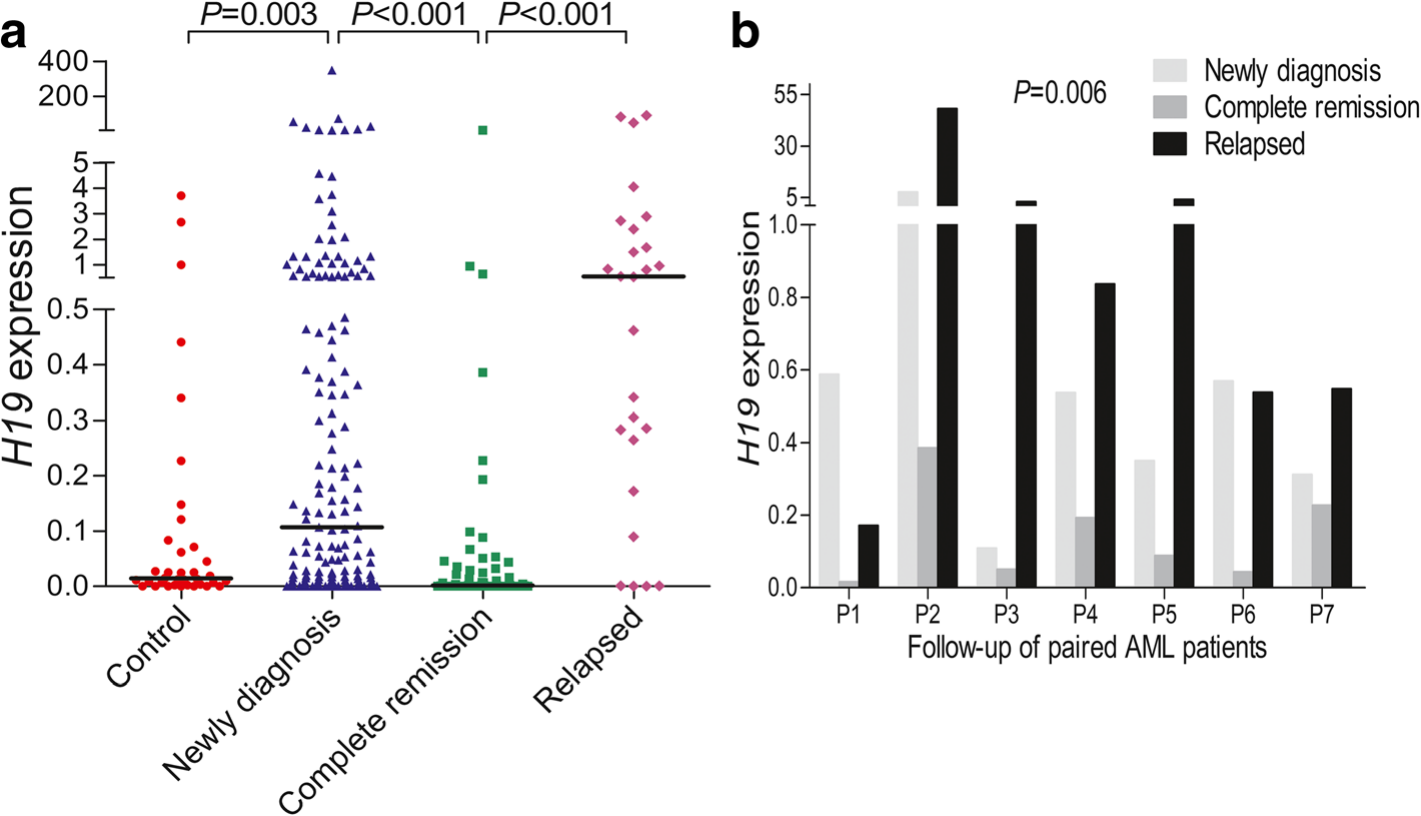
*H19* expression in the surveillance of AML. **a** *H19* expression in different clinical stages (newly diagnosis, complete remission, and relapse time) of AML patients. **b** Dynamic change of *H19* expression in the follow-up of seven paired AML patients during newly diagnosis, complete remission, to relapse time

To identify whether *H19* expression could act as a potential biomarker in the surveillance of AML, we next assessed *H19* expression in AML patients of different clinical stages including 54 patients who achieved CR after induction therapy and 26 relapsed patients. Our data indicated that *H19* expression in CR phase was lower to diagnosis time and was returned to primary level when in relapse time (Fig. 4a). Moreover, the dynamic changes of *H19* expression in seven paired patients with available follow-up data were also shown in Fig. 4b.

### *H19* exhibited pro-proliferative and anti-apoptotic effects in leukemia cells

**Fig. 5:**
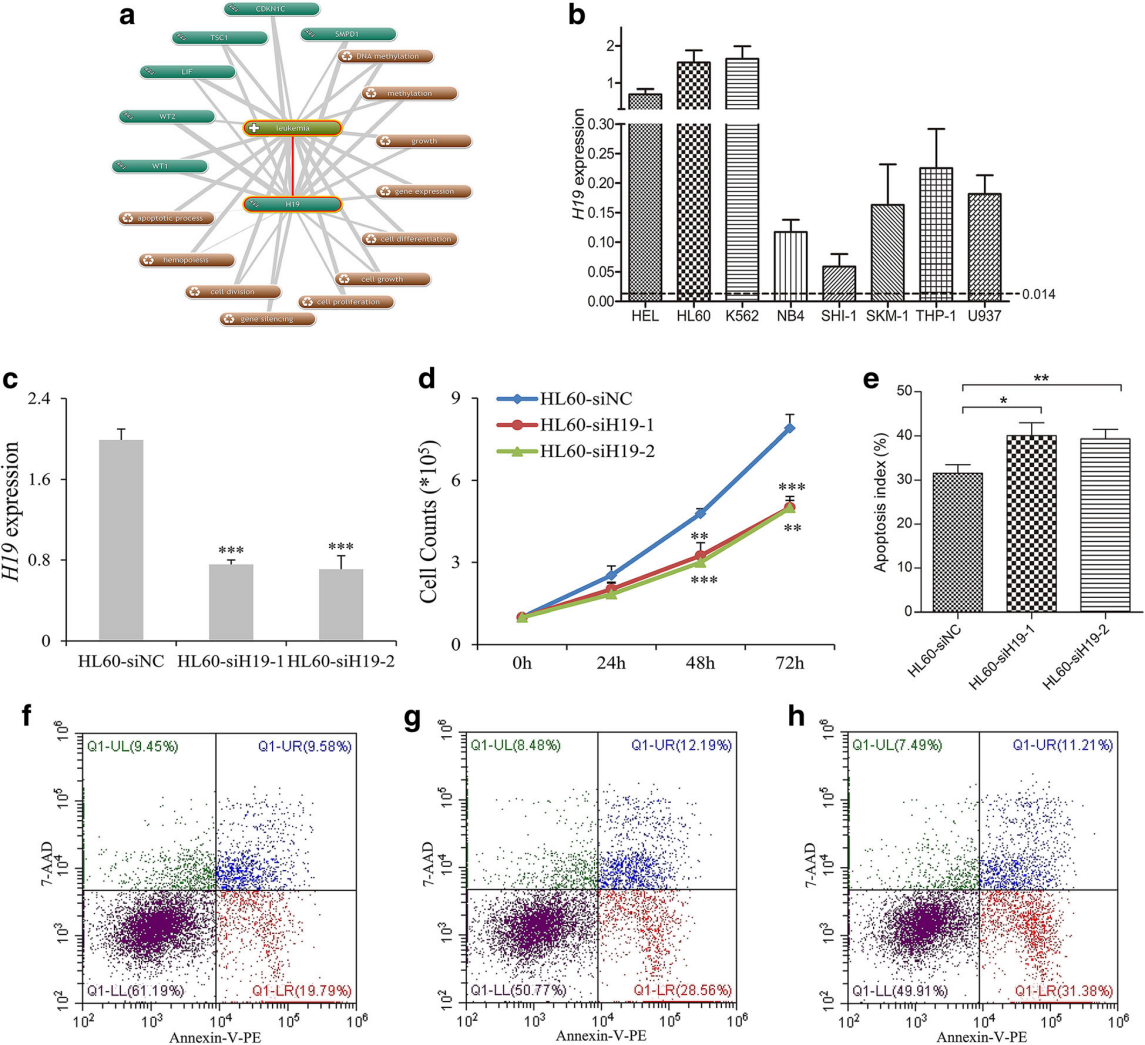
The biological role of *H19* on leukemic cell line HL60. **a** The underlying role of *H19* in leukemogenesis determined by Coremine Medical online database (http://www.coremine.com/medical/). **b** *H19* expression in eight common leukemic cell lines. The dotted line indicated the cutoff value to define *H19* overexpression. **c** *H19* expression mediated by siRNA-based *H19* knockdown. *H19* expression was significantly downregulated in siRNA-based *H19* knockdown (siH19-1 and siH19-2) and control (siNC) groups. **d** The effect of *H19* knockdown on cell proliferation. The si*H19* groups (si*H19*-1 and si*H19*-2) showed significantly lower proliferation capacity than the siNC group at 48 and 72 h. **e** The effect of *H19* knockdown on cell apoptosis. The si*H19* groups (siH19-1 and siH19-2) showed significantly higher apoptosis rate than the siNC group at 48 h. **f**–**h** Flow-type apoptosis figures for siNC, si*H19*-1, and si*H19*-2, respectively. *P < 0.05, **P < 0.01, ***P < 0.001

We first identified the potential biological role of *H19* in leukemia by bioinformatics analysis on the basis of Coremine Medical mining. As shown in Fig. 5a, the associations of *H19* with proliferation, division, differentiation, apoptotic process, and hemopoiesis were comprehensively analyzed. Next, we performed in vitro experiments to validate the leukemia-promoting effects of *H19* in AML. Since all the leukemic cells showed an increased *H19* expression, we conducted loss-of-function assays in *H19* relatively high-expressed cells (Fig. 5b, c). As a result, knockdown of *H19* in HL60 cells by two different siRNAs resulted in a significantly reduced proliferation and elevated apoptosis (Fig. 5d–g). In addition, similar results were also observed in K562 cells and had been published in our previous study [30].

### *H19* expression positively correlated with potential downstream gene ID2 in AML

**Fig. 6:**
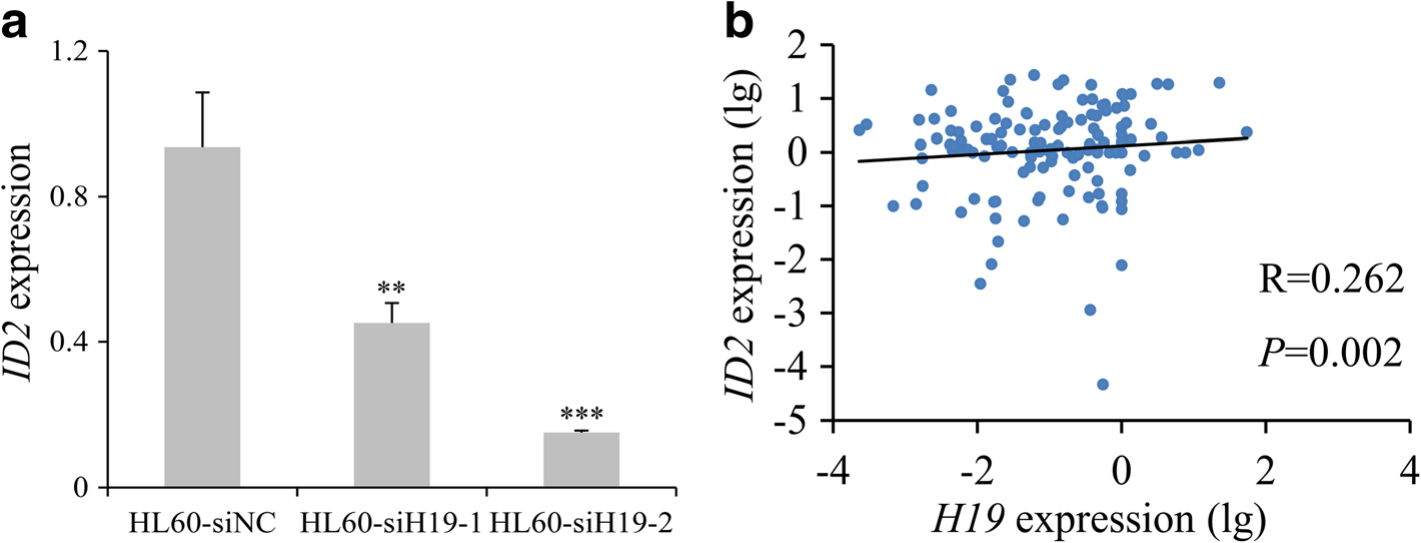
Relationship between *H19* and *ID2* in AML. **a** *ID2* mRNA expression in after siRNA-based *H19* knockdown in HL60 cell line. *ID2* mRNA expression was significantly downregulated after siRNA-based *H19* knockdown (siH19-1 and siH19-2) and control (siNC) groups. **P < 0.01, ***P < 0.001. **b** Correlation between *H19* and *ID2* expressions in AML patients. Correlation analysis was performed by Spearman test

As is well-known, lncRNAs function directly or indirectly through the protein-encoding gene. A previous study showed that *H19* was positively associated with *ID2* expression in bladder cancer [31]. Moreover, *ID2* overexpression was a frequent event and predicted poor chemotherapy response and adverse prognosis in AML [32]. Herein, we also found knockdown of *H19* also induced decreased *ID2* expression in HL60 cells (*P* = 0.006, Fig. 6a). Moreover, significant positive association was also observed between *H19* expression and *ID2* transcript level in clinical AML patients (*R* = 0.262, *P* = 0.002, *n* = 135, Fig. 6b). All these suggested that *ID2* might be a potential downstream gene of *H19* in AML.

## Discussion

Oncogenic role of lncRNA *H19* has been demonstrated in diverse human solid tumors, and *H19* expression was significantly upregulated in these cancer patients [5]. In this study, we first quantified *H19* expression in BMMNCs of AML patients and showed that *H19* overexpression was a frequent event in AML. We next performed functional experiments in vitro to investigate the potential role of *H19* in AML. Loss-of-function of *H19* by siRNA in human HL60 cells exhibited anti-proliferative and pro-apoptotic effects in accordance with previous literatures showing the role of *H19* in solid tumors [5]. In addition, a recent study showed the functional involvement of *H19* in *BCR-ABL*-mediated leukemogenesis [7]. Taken together, all these data implicated that *H19* might also act as a proto-oncogene during leukemogenesis. However, Tessema et al. showed *H19*/*IGF2* was frequently downregulated in AML, CML, and chronic myelomonocytic leukemia (CMML) [33]. One explanation for the differing results may be attributed to the limited cases of AML, CML, and CMML in the previous study.

As is well known, lncRNAs function often through promoting the strength of specific enhancer-promoter looping and thus contributing to gene activation, regulating protein activities, sequestering microRNAs, and serving as precursors of small RNAs during the pathological processes [4]. In addition to *H19*, it can be dissected into two major functions: one is a reservoir of *miR-675* that suppresses its targets, and the other is a modulator of micro-RNAs or proteins via their binding [5]. However, our study showed that *miR-675* expression was significantly downregulated in AML patients [29] and was not correlated with *H19* expression, which indicated that the function of *H19* during leukemogenesis was not mediated by *miR-675*. Notably, our study further confirmed *H19* expression was positively associated with *ID2* expression in AML. Coincidentally, a recent study reported *H19* regulated *ID2* expression through competitive binding to *hsa-miR-19a/b* in AML [34]. All these suggested that the function of *H19* may be mediated by *ID2* during leukemogenesis.

DNA methylation, one of the most common epigenetic modifications, has been related to various regulatory processes, such as transcriptional regulation, LOI, chromatin structure, and genome integrity [35]. Strong evidence has proved that aberrant *H19* DMR/ICR methylation by controlling CTCF6 binding sites led to LOI of *IGF2*/*H19* and finally resulted in abnormal expression of *IGF2*/*H19* in diverse human cancers [36–38]. Moreover, our previous study showed that *H19* DMR/ICR demethylation resulted in upregulation of *H19* expression in leukemic cell line K562 [39]. Herein, we also investigated the pattern of *H19* DMR/ICR methylation in AML patients and determined the association with *H19* expression. However, our data found that *H19* DMR/ICR methylation level was similar to controls and was not associated with *H19* expression. These results suggested that *H19* overexpression in AML was not dependent on *H19* DMR/ICR methylation. Therefore, other mechanisms were involved in the regulation of *H19* expression in AML, and further studies were urged to identify the underlying mechanism.

Clinical significance of *H19* expression was increasingly investigated in solid tumors. A recent meta-analysis showed that *H19* expression might be a novel molecular marker for predicting prognosis and could also be a predictive factor of clinicopathological features in various cancers [40]. Herein, we found that *H19* overexpression was also associated with age, WBC, karyotypic classifications, and several common gene mutations in AML patients. Moreover, *H19* overexpression also acted as an independent prognostic biomarker for OS in non-APL-AML patients, and the similar results were also confirmed by TCGA and GEO data. In addition, we further identified that *H19* expression was changed in response to chemotherapy in AML. Significantly, *H19* expression in relapsed AML patients was markedly higher than AML patients who achieved CR and newly diagnosed AML patients, which implicated that *H19* also played a role in AML recurrence. All these results indicated that *H19* was a potential therapeutic target in AML and using *H19*-based targeted therapy could improve the clinical outcome for AML patients.

## Conclusions

Our findings revealed that methylation-independent *H19* is a prognostic and predictive biomarker in AML, and *H19*/*ID2* played crucial roles in leukemogenesis with potential therapeutic target value.

## Additional files

Additional file 1: **Table S1.** Primers used for RQ-PCR, RQ-MSP, and BSP. (DOCX 16 kb)

Additional file 2: **Figure S1.** ROC curve analysis using *H19* expression for discriminating AML patients from controls. (DOCX 126 kb)

## Abbreviations

AML: Acute myeloid leukemia
AUC: Area under the ROC
BM: Bone marrow
BMMNCs: BM mononuclear cells
BSP: Bisulfite sequencing PCR
CML: Chronic myeloid leukemia
CMML: Chronic myelomonocytic leukemia
CN-AML: Cytogenetically normal AML
CR: Complete remission
DMR/ICR: Differentially methylated region/imprinting control region
GEO: Gene Expression Omnibus
HRMA: High-resolution melting analysis
LFS: Leukemia-free survival
lncRNAs: Long non-coding RNAs
LOI: Loss of imprinting
OS: Overall survival
ROC: Receiver operating characteristic
RT-qMSP: Real-time quantitative methylation-specific PCR
RT-qPCR: Real-time quantitative PCR
TCGA: The Cancer Genome Atlas
