# Supplementary material for: H19 overexpression promotes leukemogenesis and predicts unfavorable prognosis in acute myeloid leukemia
Source: Clin Epigenetics. 2018 Apr 10;10:47. doi: 10.1186/s13148-018-0486-z (PMC5891930; doi:10.1186/s13148-018-0486-z)
Supplement: Supplementary file 1 — Table S1. Primers used for RQ-PCR, RQ-MSP, and BSP. (DOCX 16 kb) [file 13148_2018_486_MOESM1_ESM.docx]

**Table S1：Primers used for RQ-PCR, RQ-MSP and BSP.**

| Primers | Primer sequence (5’to 3’) | Predicted product size (bp) |
| --- | --- | --- |
| RQ-PCR primers |  |  |
| *H19*-F | GGGTCAGACAGGGACATGG | 354 |
| *H19*-R | GAGCGGTGAGGGCATACA |  |
|  |  |  |
| RQ-MSP primers |  |  |
| *H19*-MF | GGTTTTATCGTTTGGATGGTAC | 162 |
| *H19*-MR | GTAAACCCTACGACGCGTA |  |
| *H19*-UF | GGTTTTATTGTTTGGATGGTAT | 162 |
| *H19*-UR | ATAAACCCTACAACACATA |  |
|  |  |  |
| BSP primers |  |  |
| *H19*-BF | TATGGGTATTTTTGGAGGTTTTTT | 311 |
| *H19*-BR | AAATCCCAAACCATAACACTAAAAC |  |

RQ-PCR: real-time quantitative PCR; RQ-MSP: real-time quantitative methylation-specific PCR; BSP: bisulfite sequencing PCR.
