# Supplementary figures and images for: H19 overexpression promotes leukemogenesis and predicts unfavorable prognosis in acute myeloid leukemia
Source: Clin Epigenetics. 2018 Apr 10;10:47. doi: 10.1186/s13148-018-0486-z (PMC5891930; doi:10.1186/s13148-018-0486-z)

**Figure S1. ROC curve analysis using *H19* expression for discriminating AML patients from controls.**


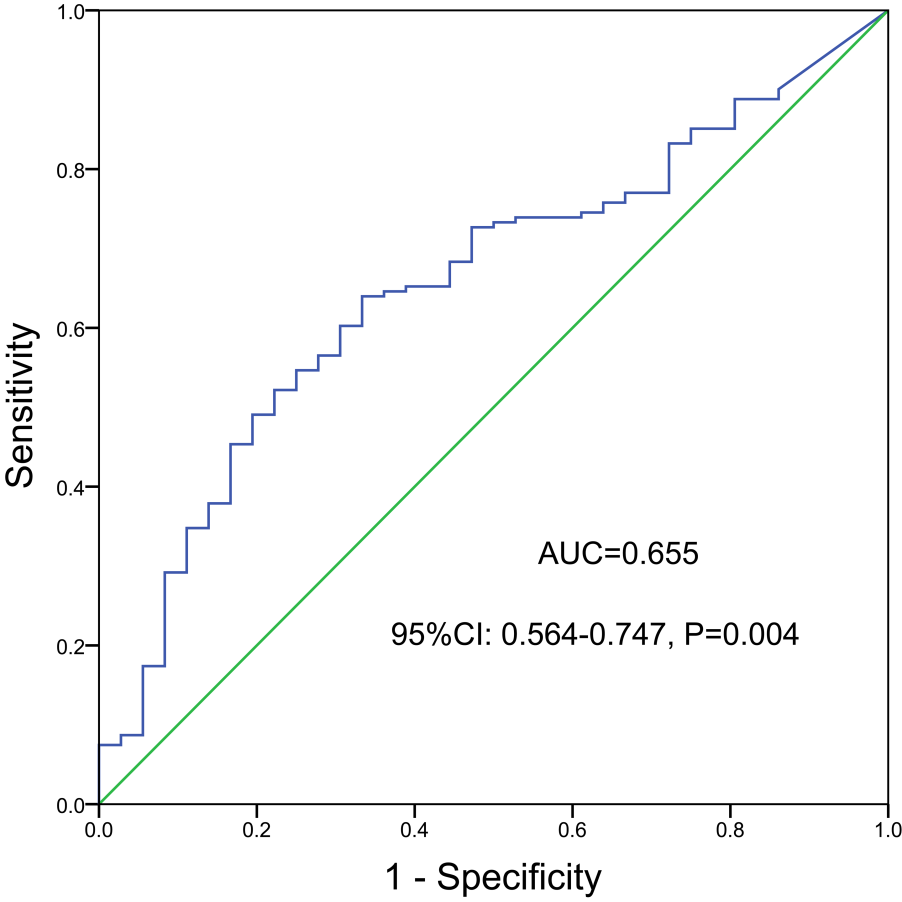

Supplement: Supplementary file 2 — Figure S1. ROC curve analysis using H19 expression for discriminating AML patients from controls. (DOCX 126 kb) [file 13148_2018_486_MOESM2_ESM.docx]
